# Keratins regulate colonic epithelial cell differentiation through the Notch1 signalling pathway

**DOI:** 10.1038/cdd.2017.28

**Published:** 2017-05-05

**Authors:** Iris A K Lähdeniemi, Julia O Misiorek, Christian J M Antila, Sebastian K-J Landor, Carl-Gustaf A Stenvall, Lina E Fortelius, Linda K Bergström, Cecilia Sahlgren, Diana M Toivola

**Affiliations:** 1 Cell Biology, Biosciences, Faculty of Science and Engineering, Åbo Akademi University, Turku, Finland; 2 Turku Center of Biotechnology, University of Turku and Åbo Akademi University, Turku, Finland; 3 Department of Cell and Molecular Biology, Karolinska Institutet, Stockholm, Sweden; 4 Technical University of Eindhoven, Eindhoven, The Netherlands; 5 Turku Center for Disease Modeling, University of Turku, Turku, Finland

## Abstract

Keratins (K) are intermediate filament proteins important in stress protection and mechanical support of epithelial tissues. K8, K18 and K19 are the main colonic keratins, and K8-knockout (K8^−/−^) mice display a keratin dose-dependent hyperproliferation of colonic crypts and a colitis-phenotype. However, the impact of the loss of K8 on intestinal cell differentiation has so far been unknown. Here we show that K8 regulates Notch1 signalling activity and differentiation in the epithelium of the large intestine. Proximity ligation and immunoprecipitation assays demonstrate that K8 and Notch1 co-localize and interact in cell cultures, and *in vivo* in the colonic epithelial cells. K8 with its heteropolymeric partner K18 enhance Notch1 protein levels and activity in a dose dependent manner. The levels of the full-length Notch1 receptor (FLN), the Notch1 intracellular domain (NICD) and expression of Notch1 downstream target genes are reduced in the absence of K8, and the K8-dependent loss of Notch1 activity can be rescued with re-expression of K8/K18 in K8-knockout CRISPR/Cas9 Caco-2 cells protein levels. *In vivo*, K8 deletion with subsequent Notch1 downregulation leads to a shift in differentiation towards a goblet cell and enteroendocrine phenotype from an enterocyte cell fate. Furthermore, the K8^−/−^ colonic hyperproliferation results from an increased number of transit amplifying progenitor cells in these mice. K8/K18 thus interact with Notch1 and regulate Notch1 signalling activity during differentiation of the colonic epithelium.

Keratins (K) are the main intermediate filament proteins of epithelial cells where they have stress-protective roles in mechanical and non-mechanical functions.^1^ Keratins bind to desmosomes between epithelial cells maintaining tissue barriers and cell polarity, participate in the spatial organization of organelles and proteins^2, 3, 4^ and in the regulation of apoptosis and cell migration.^5, 6^ In humans, K5 and K14 mutations lead to skin diseases, and mutations in K8, K18 or K19 predispose to liver diseases.^6, 7, 8^ Keratins are dynamically regulated by post-translational modifications where site-specific phosphorylation of, for example, K8 Serine (S) 74 is associated with mitosis, apoptosis and cell stress.^9^

In the colonic epithelium, the main keratin family members expressed are type II K7 and K8 that with type I K18-K20 form filaments by obligate type I–type II heteropolymerization.^10^ K8 is the most common type II keratin in the intestine, and K8^−/−^ mouse colonocytes consequently lack most keratins.^11, 12^ The K8^−/−^ mouse develops a colonic phenotype consisting of epithelial hyperproliferation, decreased apoptosis, altered chloride/sodium transport, diarrhea and a Th2-type ulcerative colitis, which is ameliorated with antibiotics.^4, 12, 13, 14, 15^ Heterozygote K8^+/−^ mice have intermediate levels of keratins compared to K8^+/+^ and K8^−/−^, and display an intermediate colonic phenotype with increased hyperproliferation and altered ion transport, but do not develop colitis.^4, 11, 15^ The influence of keratins on colonic epithelial differentiation has not been investigated, but the K8^−/−^ hyperproliferation proposes a role for keratins in the balance of colonic epithelial differentiation and cell fate.

Renewal of the colonic epithelium is a continuous and tightly controlled process. Colonic epithelial stem cells located in the crypt bottom proliferate to form transit amplifying cells, which migrate towards the top of the crypt and differentiate into enterocytes, goblet cells and enteroendocrine cells (EEC).^16^ The main regulators of colonic epithelial cell differentiation are the Notch1, Wnt and Ephrin signalling pathways.^17^ Other pathways including bone morphology protein, hedgehog and growth factor signalling also have roles in renewal of the colonic epithelium.^17^ In the Notch1 pathway, a ligand binds the full-length Notch1 receptor (FLN) on an adjacent cell, inducing cleavage of the receptor generating the membrane-tethered ΔE-Notch form, which is subsequently cleaved by the *γ*-secretase complex to release the Notch1 intracellular domain (NICD).^18, 19^ When released, NICD translocates to the nucleus and activates target gene transcription (for example *Hey1*, *Hey2*), which induces differentiation of transit amplifying cells towards enterocytes and inhibits the formation of goblet cells and EEC.^19, 20, 21, 22, 23, 24^ It is poorly understood which molecular factors regulate Notch1 signalling and colonic epithelial cell differentiation. Here we report a novel interaction between keratins and Notch1, where K8/K18 increase Notch1 levels and enhance Notch1 signalling activity. We also demonstrate that the lack of K8 *in vivo* leads to decreased Notch1 levels and signalling activity associated with a shift in colonic epithelial cell differentiation towards a goblet cell phenotype.

## Results

### K8 interacts and co-localizes with Notch1

**Figure 1:**
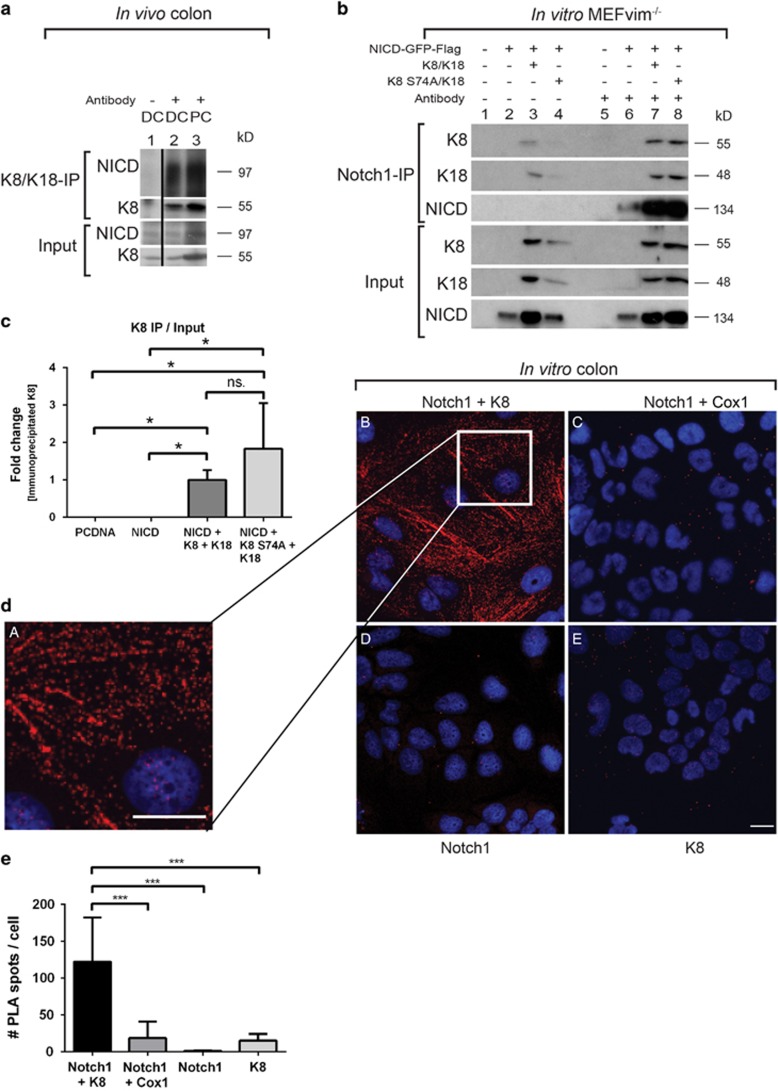
K8 binds to and co-localizes with Notch1 in immunoprecipitation and PLAs. (**a**) Proximal (PC) and distal (DC) parts of the colon epithelium were isolated by scraping and homogenized with immunoprecipitation lysis buffer. For K8/K18 immunoprecipitation, the lysates were precleared with protein-G/Sepharose beads and incubated overnight with beads and K8/K18 antibodies. The immunoprecipitates were analysed with SDS-PAGE and immunoblotting with the indicated antibodies. Input samples were collected before the immunoprecipitation. The black vertical line in the figure indicates that empty wells have been cut out from the immunoblot without affecting the horizontal level of the bands (full blots are presented in Supplementary Figure S1A). Separate negative control samples where no antibody was added (–antibody) were prepared from the same DC sample that was used for immunoprecipitation and treated the same way as the other samples except for the omission of antibody. The input results shown in the DC–antibody sample in lane 1 are the same input sample western blot result as in the DC sample, lane 2. *n*=4. (**b**) Cells were transfected with the indicated plasmids, and the samples were collected and lysed for Notch1 immunoprecipitation and input (Input) as described in (a) 24 h after transfection. The no antibody controls (lanes 1–4) and the precipitates (lanes 5–8) were precleared and incubated overnight with either beads alone or beads+goat anti-full length Notch1 c-20 antibody recognizing all Notch forms. The immunoprecipitates were analysed with SDS-PAGE and immunoblotting with the indicated antibodies (rabbit anti-full length Notch c-20 for Notch1), *n*=6. (**c**) K8 protein levels from immunoprecipitates (in (b)) were quantified and compared to the K8 input signals. The no antibody signal was subtracted from the immunoprecipitate signals and normalized to input levels. Negative values for PCDNA and NICD were set to 0. *n*=3. *P<0.05. (**d**) PLA was performed for Notch1 and K8 (dA, and which is zoomed from dB) in Caco-2 cells fixed with methanol and acetone at −20 °C. The PLA signal (red) for Notch1 and K8 was analysed using rabbit anti-full length Notch1 c-20 (FLN and NICD) and mouse anti-K8 antibody in the proximity ligation kit from Duolink. Negative controls used were PLA signals for Notch1 and Cox1 (**C**), and background signals for Notch1 (**D**) and K8 (**E**) alone. Nuclei are presented in blue. *n*=3. Scale, 20 μm. (**e**) Quantification of PLA spots per cell in (d) was performed using particle analysis in Fiji. *n*=5–13 cells/ analysis. ***P<0.001

Keratins function as scaffolds regulating the activity and localization of proteins.^6^ To explore the possible role for keratins in the regulation of colonic epithelial homeostasis, K8/K18 immunoprecipitation was performed to analyse if K8/K18 interact with known determinants of differentiation in the colon. NICD was co-immunoprecipitated in a complex with K8/K18 from murine distal and proximal colon indicating that these proteins interact (Figure 1a and Supplementary Figure S1A). An antibody recognizing all forms of Notch1 was used to immunoprecipitate Notch1 from mouse embryonic fibroblasts lacking vimentin (MEFvim^−/−^) and overexpressing NICD-GFP-Flag, ΔE Notch1 or FLN, with and without K8/K18, in order to confirm the binding and analyse which domain of Notch1 K8/K18 bind to. Western blot analysis revealed that K8 and K18 were co-immunoprecipitated from cells expressing NICD and the other Notch1 constructs (Figures 1b, c and Supplementary Figure S1B). These data support that K8/K18 interact with Notch1 at the NICD domain present in all constructs,^18, 19^ as the NICD domain alone co-immunoprecipitated K8 (Figures 1b and c). The phosphodeficient mutant protein K8 S74 to Alanine (A)^9^ also co-immunoprecipitated with Notch1 (Figure 1b, lane 8 and c), indicating that the Notch–K8 binding is not K8 S74 phosphorylation dependent. Supportive of these data, is that epithelial human embryonic kidney HEK 293 cells that overexpress FLN (HEK FLN 293),^25^ and that also express K8/K18, co-immunoprecipitated FLN with a K18 antibody (Supplementary Figure S1C).

To further analyse the spatial relationship between K8 and Notch1, proximity ligation assay (PLA) was performed using K8 and Notch1 antibodies in Caco-2 human colorectal cancer cells. The PLA signal showed that Notch1 and K8 are closely localized in PLA-positive dots both at the cell membrane and in the cytosol in filamentous arrays (Figure 1dA–B and e). PLA assay with cyclooxygenase 1 (Cox1) (negative control) and Notch1 (Figure 1dC), or with Notch1 (Figure 1dD) or K8 (Figure 1dE) alone showed no or minor PLA signal, indicating that the K8/Notch1 proximity signal is specific (Figure 1e). The co-localization of Notch1 and K8 in Caco-2 cells was confirmed by double-immunofluorescence staining, using the rabbit-Notch1 C-20 Santa Cruz antibody (also used for PLA), recognizing FLN and all cleaved fragments of Notch1, and a rat-K8 antibody (Troma I). The staining showed that Notch1 co-localizes with typical K8-filaments in the cytosol (Supplementary Figure S2A), and forms filamentous patterns also when not stained for K8 (Supplementary Figure S2C). Similar Notch1/K8 patterns were seen in a different epithelial cell line, MCF7 breast cancer cells, using different K8 (273) and Notch antibodies (A6, Supplementary Figure S2B). Our observations demonstrate that K8 co-localizes and interacts with Notch1 both *in vivo* and *in vitro* in cell culture conditions.

### Keratins enhance Notch1 levels and stabilize signalling activity *in vitro*

**Figure 2:**
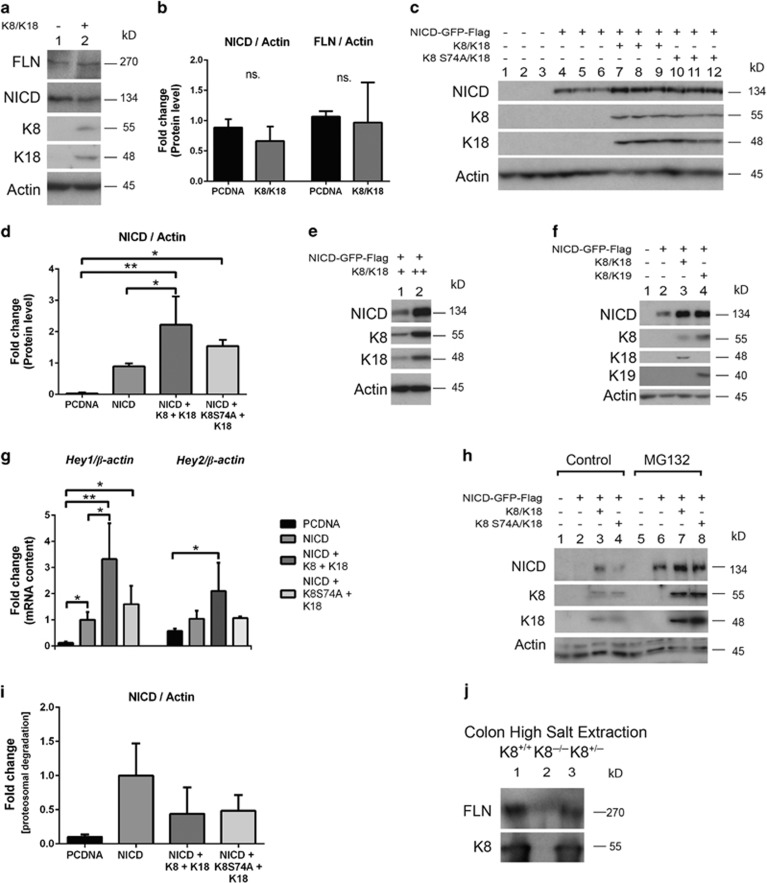
Keratins enhance and stabilize Notch levels. (**a**–**d**) MEFvim^−/−^ cells were cultured and transfected by electroporation with the indicated plasmids. FLN, NICD and K8/K18 protein levels were analysed with SDS-PAGE and immunoblotting. Actin was used as a loading control. The endogenous protein amounts of FLN and NICD from (**a**) were quantified in (**b**), and from (**c**) were quantified in (**d**) by normalization to the loading control actin. Lanes 1–12 in (**c**) represent the different cell samples (*n*=3). Lane 1 in (**a**) and lanes 1–3 in (**c**) represent transfection with an empty plasmid (PCDNA3.1). *P<0.05, ** *P*<0.01. (**e**) MEFvim^−/−^ cells were cultured and transfected by electroporation with 10 μg NICD and 5 (+) and 10 (++) μg of K8 and K18. NICD and K8/K18 protein levels were analysed by immunoblotting. Actin was used as a loading control. *n*=3. (**f**) MEFvim^−/−^ cells were cultured and transfected and analysed by western blotting as in (**c**) with the addition of K19. Actin was used as a loading control. Lane 1 represents transfection with an empty plasmid (PCDNA3.1). *n*=4. (**g**) MEFvim^−/−^ cells were cultured and transfected as indicated in (**c**) and *Hey1* and *Hey2* mRNA amounts were analysed with RT-PCR, normalized to *β-Actin* and presented as average fold change as related to NICD overexpressed control cell samples±SD. *n*=3. *P<0.05. **P<0.01. (**h**) MEFvim^−/−^ cells were transfected by electroporation of the indicated plasmids. 24 h after transfection, cells were treated for 12 h with 20 μM MG132. Cell lysates were analysed with SDS-PAGE and immunoblotting using the indicated antibodies. Actin was used as a loading control. Lanes 1 and 5 represent transfection with an empty plasmid (PCDNA3.1), *n*=3. (**i**) The NICD levels in (**h**) were normalized to the loading control actin, whereafter the NICD levels in MG132 treated cells were normalized to control cells to obtain average fold change proteosomal degradation. Data are presented as average fold change and related to NICD-overexpressed control cell samples±S.D. *n*=3. **(j)** Colon from K8^+/+^, K8^−/−^ and K8^+/+^ mice were subjected to high salt extraction, and the cytoskeletal fraction was analysed by SDS-PAGE and immunoblotting for FLN and K8

The interaction of Notch1 with K8 indicates a role for K8 in Notch1 regulation. We tested this hypothesis by investigating the influence of K8/K18 on Notch1 protein levels in MEFvim^−/−^ cells. Overexpression of K8/K18 in MEFvim^−/−^ cells did not affect the endogenous FLN or NICD levels in this fibroblast cell line (Figures 2a and b). In order to test whether K8/K18 can stabilize NICD levels in an artificial situation, we transfected cells with NICD together with K8/K18. NICD levels were consistently 2–3 fold higher when overexpressing NICD with K8/K18 compared with NICD alone (Figures 2c and d), and the NICD levels increased in a keratin-dose-dependent manner (Figure 2e). Overexpression of phosphorylation-deficient K8 S74A was also able to increase NICD levels (Figures 2c and d). In addition, overexpression of K8 with a different keratin partner, K19, with NICD, similarly to K8/K18 increased the NICD levels indicating that the NICD increase is K8-dependent but independent of its heterodimeric partner (Figure 2f). In order to test whether K8 affects Notch1 signalling levels, the effect of keratin overexpression in MEFvim^−/−^ cells on Notch1 target genes was analysed. Expression of the Notch1 target genes *Hey1* and *Hey2* was significantly increased when NICD was overexpressed together with K8/K18 compared to NICD overexpression alone (Figure 2g). Overexpression of K8 S74A/K18 with NICD did not increase the mRNA levels of *Hey1* or *Hey2* (Figure 2g) suggesting that phosphorylation of K8 S74 may have a role in the regulation of Notch signalling activity. This is in contrast to the influence of keratin phosphorylation on Notch binding and NICD protein levels (Figure 1b, 2c, d and h). To determine whether K8/K18 stabilize NICD, the proteasome was inhibited with MG132 for 12 h. A difference in NICD levels in cells expressing NICD and in cells expressing NICD with K8/K18 or K8 S74A/K18 could not be observed (Figures 2h and i), suggesting that keratins do not significantly affect the degradation speed of NICD. To inhibit protein translation, MEFvim^−/−^ cells overexpressing NICD alone or together with K8/K18 or K8 S74A/K18 were treated for 0, 3 and 6 h with cycloheximide. Although the relative K8/K18-induced NICD levels decreased successively with a similar turnover rate as for NICD alone (Supplementary Figure S3A and C), the actual levels of NICD were consistently 2–3 fold higher if K8/K18 or K8 S74A/K18 were present (Supplementary Figure 3A and B). Similarly, to test the turnover rate of FLN, Caco-2 cells where K8 had been knocked out using the CRISPR/Cas9 method were used.^26^ The turnover rate of endogenous FLN was similar in the presence or absence of keratins (Supplementary Figure S3D). In order to determine whether K8/K18 can affect the Notch subcellular localization, high salt extractions from K8^+/+^ and K8^−/−^ colon were analysed, and both FLN and NICD were detected in the high salt cytoskeleton fraction (Figure 2j). Together, these data suggest that keratins enhance Notch1 levels and signalling activity.

### Notch1 levels and signalling activity are decreased in the K8^−/−^ mouse colonic epithelium

**Figure 3:**
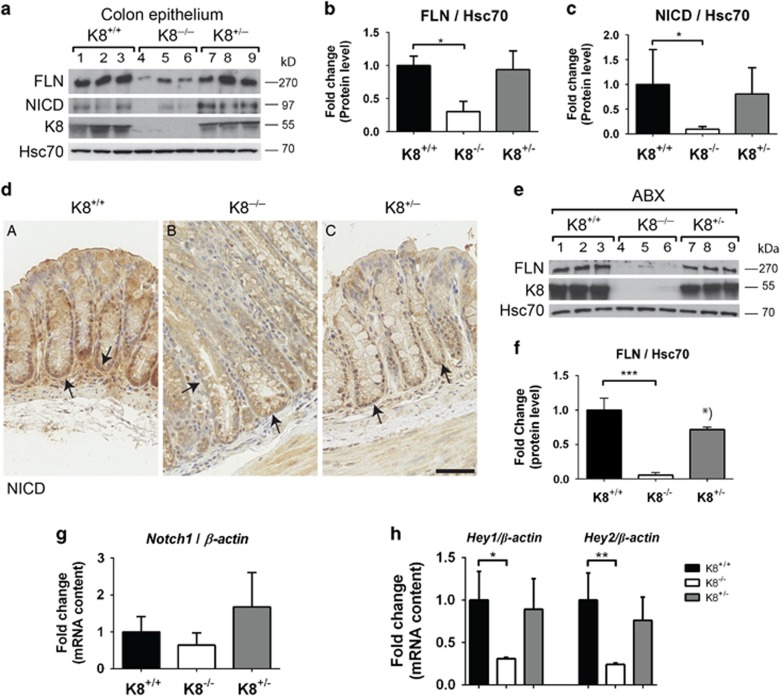
FLN and NICD are decreased in K8^−/−^ mouse colon. (**a**) Colonic epithelium isolated by scraping was used for analysis of FLN and NICD protein amounts with SDS-PAGE and immunoblotting. The genotypes (K8^+/+^, K8^−/−^ and K8^+/−^) were confirmed by K8 immunoblotting, and Hsc70 was used as a loading control. Lanes 1–9 represent individual mice. *n*=3. (**b** and **c**) The protein amount of FLN and NICD from (**a**) was quantified and normalized to the loading control Hsc70. *n*=3. The results are presented as average fold change as related to K8^+/+^ control mice±SD. *P<0.05. (**d**) Immunohistochemical staining for NICD was performed on paraffin-embedded colon sections using rabbit anti-cleaved Notch1 antibody as indicated. *n*=3. Scale, 50 μm. Arrows point to positive signal in the crypt regions. (**e**) Mice were treated with broad-spectrum antibiotics (ABX) for 8 weeks after which the protein levels of FLN and K8 were analysed with SDS-PAGE and immunoblotting in colonic epithelial scraping samples. Hsc70 was used as a loading control. *n*=3. (**f**) FLN protein levels from (**e**) were quantified, normalized against the loading control Hsc70 and presented as average fold change as related to K8^+/+^ control mice±SD. ***P<0.001.*) K8^+/−^ mice did not show a significant difference in FLN levels compared to K8^+/+^ by one-way Anova, whereas with Student's *t*-test the *P* value was 0.05. (**g**) *Notch1* mRNA levels were analysed with RT-PCR in the indicated mouse genotypes, normalized to *β-Actin* mRNA and presented as average fold change±SD. *n*=3. (**h**) mRNA levels of Notch target genes *Hey1* and *Hey2* were analysed with RT-PCR in the indicated mouse genotypes, were normalized to *β-Actin* and presented as average fold change as related to K8^+/+^ control mice±SD. *n*=3. *P<0.05, **P<0.01

We next analysed Notch1 signalling activity in the K8^−/−^, K8^+/+^ and K8^+/−^ mouse colonic epithelium *in vivo*. At the protein level, both FLN and NICD were significantly decreased more than threefold in K8^−/−^ mice (Figures 3a–c) compared to K8^+/+^ mice. Immunohistochemical staining using an antibody against the NICD-cleaved epitope (recognizing the active NICD), confirmed that NICD was decreased in the K8^−/−^ crypt (Figure 3dB). Colonic luminal bacteria can directly activate Notch1 target genes and differentiation.^27^ To verify that the Notch1 phenotype was a consequence of the loss of keratins and not of bacteria or inflammation, we treated mice with antibiotics to eliminate the intestinal microflora and ameliorate the K8^−/−^ colitis.^13^ The K8^−/−^ Notch1 phenotype was not dependent on intestinal bacteria and colonic inflammation as mice treated with broad-spectrum antibiotics for 8 weeks^13, 14^ showed a similar robust decrease in K8^−/−^ FLN protein levels compared to K8^+/+^ mice (Figures 3e and f) as untreated mice (Figures 3a and b). The FLN levels in K8^+/−^ mice, which do not have colitis, were not affected under basal conditions, but displayed a mild decrease after antibiotics treatment compared to K8^+/+^ (Figures 3e and f). The decrease was not statistically significant using one-way Anova analysis, but reached a *P*-value of 0.05 with Student's *t*-test. *Notch1* mRNA levels remained unaltered in the K8^−/−^ and K8^+/−^ colon (Figure 3g). In accordance with the reduced Notch1 protein levels in untreated mouse colon (Figures 3a and b), expression of the Notch1 downstream target genes *Hey1* and *Hey2* was significantly decreased in K8^−/−^ colon (Figure 3h).

### Notch1 levels and signalling activity are decreased in CRISPR/Cas9 K8^−/−^ cells and rescued by K8/K18 re-expression

**Figure 4:**
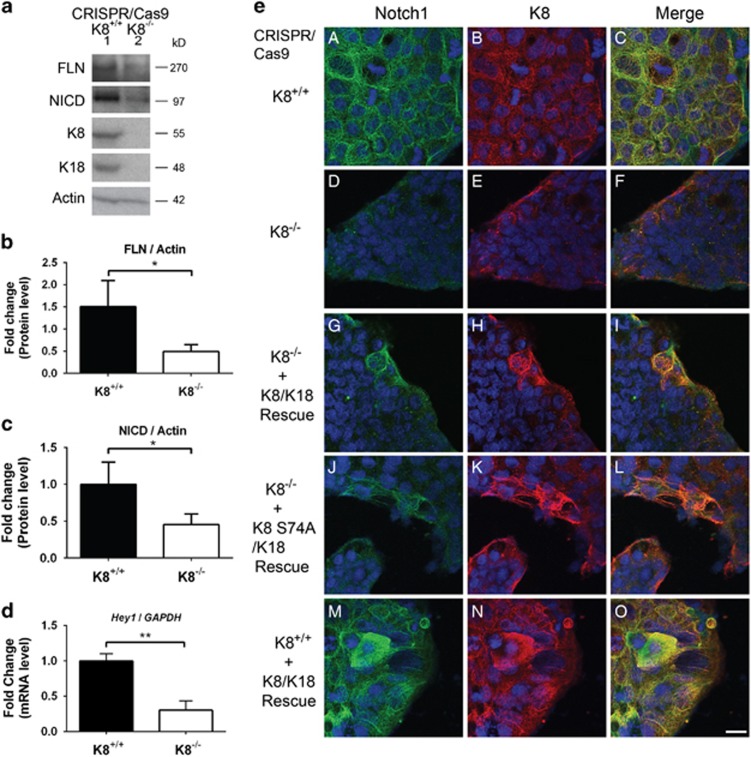
K8 deletion with the CRISPR/Cas9 method in Caco-2 cells downregulates Notch1 levels, which are rescued by re-expression of K8/K18 or K8 S74A/K18. (**a**) Caco-2 CRISPR/Cas9 K8^+/+^ and K8^−/−^ cells were cultured, lysed for protein analysis and analysed with the indicated antibodies. Actin was used as a loading control. *n*=5. (**b** and **c**) The FLN and NICD protein levels in (**a**) were quantified and normalized to the loading control actin. *n*=3. The results are presented as average fold change as related to K8^+/+^ control cells±SD. * *P*<0.05. (**d**) Notch target gene Hey1 was analyzed in Caco-2 CRISPR/Cas9 K8^+/+^ and K8^−/−^ cells with RT-PCR, normalized to β-Actin mRNA and presented as average fold change ± S.D. *n* = 3. (**e**) Caco-2 CRISPR/Cas9 K8^+/+^ (A–C, M–O) and Caco-2 CRISPR/Cas9 K8^−/−^ (D–L) cells were cultured on cover slips, and K8/K18 or K8 S74A/K18 were overexpressed in Caco-2 CRISPR/Cas9 K8^−/−^ (G–L), and K8/K18 in Caco-2 CRISPR/Cas9 K8^+/+^ (M–O) cells with Lipofectamine 2000. Cells were fixed with methanol and acetone in −20 °C and immunostained for Notch1 (A, D, G, J, M) and K8 (B, E, H, K, N) and showed separately, or merged (C, F, I, L, O). Nuclei are presented in blue. *n*=3–6. Scale, 20 μm

**Figure 5:**
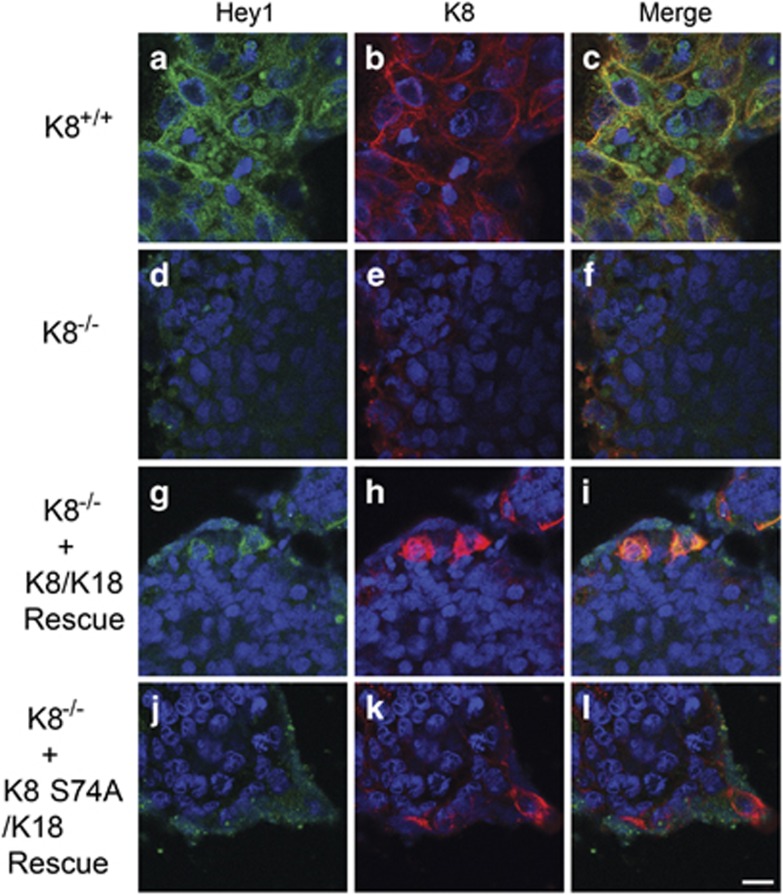
K8 deletion with the CRISPR/Cas9 method in Caco-2 cells downregulates Hey1 levels, which are rescued by re-expression of K8/K18. Caco-2 CRISPR/Cas9 K8^+/+^ (**a**–**c**) and Caco-2 CRISPR/Cas9 K8^−/−^ (**d**–**l**) cells were cultured on cover slips, and K8/K18 or K8 S74A/K18 were overexpressed in Caco-2 CRISPR/Cas9 K8^−/−^ (**g**–**l**) cells with Lipofectamine 2000. Cells were fixed with methanol and acetone in −20 °C and immunostained for Hey1 (Hrt1, Santa Cruz; **a**, **d**, **g**, **j**) and K8 (**b**, **e**, **h**, **k**) and showed separately, or merged (**c**, **f**, **i**, **l**). Nuclei are presented in blue. *n*=3. Scale, 20 μm

To test whether K8 can stabilize endogenous Notch1 levels in the context of an epithelial colonic cell line, we analysed FLN and NICD levels and activity in CRISPR/Cas9 K8^−/−^ cells. K8-knockdown also leads to a deletion of K18 as K18 is degraded without its heteropolymeric partner K8.^28, 29^ Similar to the K8^−/−^ mouse colon (Figure 3a), both FLN and NICD levels were decreased in CRISPR/Cas9 K8^−/−^ cells compared to those in K8^+/+^ cells (Figures 4a–c). Hey1 mRNA levels were, consequently, 0.3-fold decreased in CRISPR/Cas9 K8^−/−^ compared to control K8^+/+^ cells (Figure 4d). This was further confirmed with immunofluorescence staining, indicating a dramatic loss in Notch1 after K8 deletion (Figure 4eD–F) compared to control CRISPR/Cas9 Caco-2 cells with keratins (Figure 4eA–C). The Notch1 phenotype could be rescued by overexpressing K8/K18 or K8 S74A/K18 in the CRISPR/Cas9 K8^−/−^ cells (Figure 4eG–L; see also Supplementary Figures S2C–D). Overexpression of K8/K18 in CRISPR/Cas9 K8^+/+^ cells further increased Notch1 levels (Figure 4eM–O) compared to that in untransfected K8^+/+^ cells (Figures 4A–C). To test whether cells rescued with K8/K18 have functional NICD, cells were stained for Hey1. Similar to Notch levels (Figure 4), Hey1 levels were decreased in CRISPR/Cas9 K8^−/−^ cells (Figures 5d–f) compared to K8^+/+^ cells (Figures 5a–c). The Hey1 phenotype could further be rescued by overexpression of K8/K18 in K8^−/−^ cells (Figures 5g–i) indicating that Notch signalling is active in cells rescued with K8/K18. The rescue of Hey1 was not obvious with K8 S74A/K18 (Figures 5j–l) indicating, similarly to the *Hey1* mRNA results (Figure 2g), that the keratin-dependent Notch1 activation of its target genes may be dependent on K8 phosphorylation. As the K8-dependent Notch1 phenotype was rescued in a dose-dependent manner with K8/K18 overexpression, the data suggest that keratins modulate Notch1 protein levels and signalling activity *in vivo* in the mouse colonic epithelium and in Caco-2 colonic epithelial cells.

### The lack of keratins leads to altered colonic epithelial cell differentiation towards a secretory cell fate

**Figure 6:**
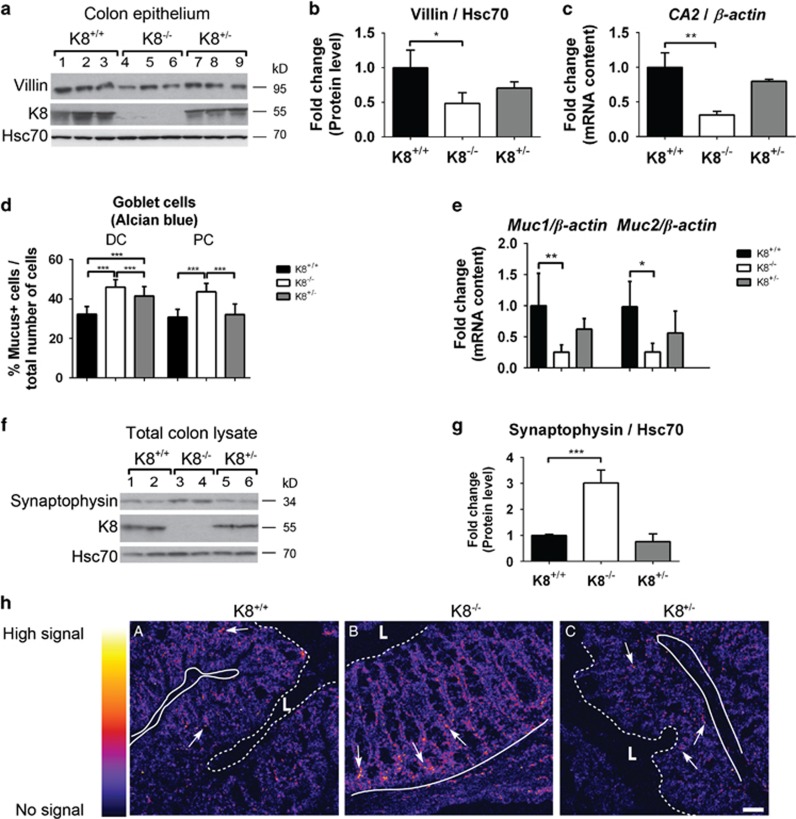
The colonic differentiation phenotype in K8^−/−^ colonic epithelium is shifted towards increased goblet cells and EEC. (**a**) Colon epithelium was isolated by scraping and the protein levels of villin were analysed with SDS-PAGE and immunoblotting. Hsc70 was used as a loading control. Lanes 1–9 represent individual mice. *n*=3. (**b**) The villin protein levels in (**a**) were normalized to the loading control Hsc70. *n*=3. The results are presented as average fold change as related to K8^+/+^ control mice±SD. *P<0.05. (**c**) *CA2* mRNA was analysed in mouse colonic epithelium with RT-PCR in the indicated mouse genotypes and normalized to *β-Actin*. The results are presented as average fold change as related to K8^+/+^ control mice±SD. *n*=3. **P<0.01. (**d**) Mucus, marking the intestinal goblet cells, was stained with Alcian blue (Supplementary Figure S4), and the mucus positive (mucus+) goblet cells were quantified by dividing the number of goblet cells with the total number of epithelial cells per colon crypt. *n*[DC]= 3 mice (10 crypts/mouse), *n*[PC]= 3 mice (10 crypts/mouse). ***P<0.001. (**e**) Goblet cell mRNA products *Mucin1 (Muc1)* and *Mucin2 (Muc2)* were analysed with RT-PCR in the indicated mouse genotypes and were normalized to *β-Actin*. *n*[*Mucin1*]= 5, *n*[*Mucin2*]= 4. The results are presented as average fold change as related to K8^+/+^ control mice±SD. *P<0.05. **P<0.01. (**f**) Colon lysates were analysed for the EEC marker synaptophysin using SDS-PAGE and immunoblotting. Hsc70 was used as a loading control. Lanes 1–6 represent individual mice. *n*=3. (**g**) The protein amount of synaptophysin from (**f**) was quantified and normalized to the loading control Hsc70. The results are presented as average fold change as related to K8^+/+^ control mice±SD. *n*=3. ***P<0.001. (**h**) Frozen colonic sections were fixed with 4% PFA and immunostained for synaptophysin. A heat map of the positive synaptophysin staining was constructed using BioImage XD. The dark colour represents no signal intensity and the light signal represents high signal intensity. The arrows point to examples of EEC. The border between the lumen (**L**) and the top of the epithelium is indicated with a dotted line and the base of the epithelial crypts by a solid line. Scale, 100 μm

To test whether K8 deletion affects the colonic epithelial cell differentiation in accordance with its effect on Notch1, we analysed EEC, enterocytes and goblet cells in the crypt. The Notch1 signalling pathway is the main regulator of colonic epithelial cell differentiation inducing enterocyte differentiation while inhibiting goblet cell and EEC differentiation.^20, 21, 22, 23^ The cell composition and/or pattern in the epithelial layer was analysed using the brush-border protein villin and carbonic anhydrase 2 (CA2) as markers for enterocytes,^30^ mucus as a marker for goblet cells^20^ and synaptophysin as a marker for EEC.^31^ Villin levels were decreased in the K8^−/−^ colon compared to K8^+/+^ and K8^+/−^ (Figures 6a and b), suggesting a reduced number of enterocytes in K8^−/−^ mouse colon. The enterocyte secretion product *CA2*^20^ mRNA levels were also significantly decreased in K8^−/−^ mice (Figure 6c), supporting a reduced number of enterocytes. K8^+/−^ enterocyte markers were partially, but consistently, decreased, but did not reach statistical difference (Figures 6a–c).

Alcian blue staining highlights acidic mucus in intestinal goblet cells and can be used to analyse the number of goblet cells.^32^ Quantification (Figure 6d) of Alcian blue staining (Supplementary Figure S4) demonstrated that the number of goblet cells in relationship to the total number of cells in a crypt was significantly increased in both K8^−/−^ and K8^+/−^ distal colon and K8^−/−^ proximal colon, but not in K8^+/−^ proximal colon compared to K8^+/+^ (Figure 6d). Staining of goblet cells with periodic acid Schiff (PAS) showed similar results (not shown). The mRNA levels of the goblet cell products *Mucin1* and *Mucin2*^33^ were decreased in K8^−/−^ colon (Figure 6e), suggesting that also the terminal differentiation of goblet cells may be disrupted. Thereby, our results demonstrated that K8^−/−^ and K8^+/−^ have an increased differentiation towards the secretory goblet cell fate.

Colonic EECs were analysed by immunoblotting using synaptophysin as a marker, and the levels in the K8^−/−^ mouse colon were increased compared to K8^+/+^ (Figures 6f and g). Immunofluorescence staining confirmed the increased number of synaptophysin-positive EEC cells which are concentrated to the K8^−/−^ crypt bottom (Figure 6hB), in contrast to the lower EEC numbers in K8^+/+^ (Figure 6hA) and K8^+/−^ (Figure 6hC) colon. Taken together, our results show decreased protein levels of villin, and increased number of goblet cells and levels of synaptophysin in K8^−/−^ and partly in K8^+/−^, which indicates that keratins might modulate lineage specification in colonic crypts in a dose-dependent manner through Notch1.

### K8 inactivation leads to a widened proliferative zone and an increased number of transit amplifying cells in the mouse colon

**Figure 7:**
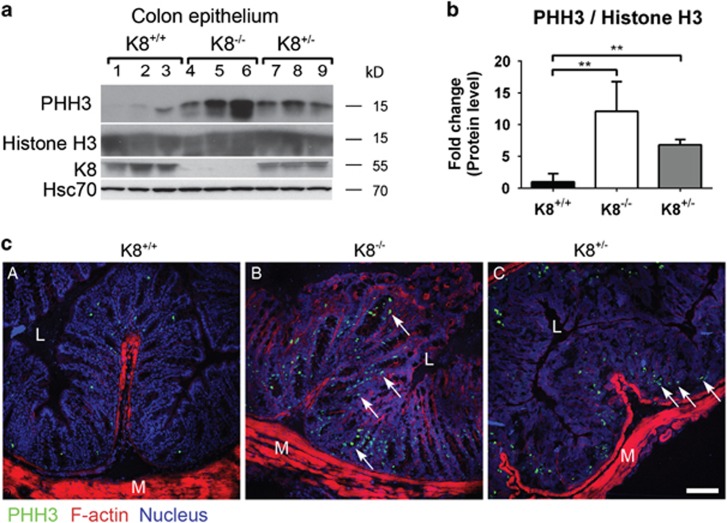
Deletion of K8 leads to an increased amount of transit amplifying cells in the colonic epithelium. (**a**) Colonic epithelium isolated by scraping was analysed with SDS-PAGE and immunoblotting for the indicated proteins. PHH3 was used as a marker of colonic epithelial transit amplifying cells. The genotypes were confirmed by K8 immunoblotting, and Hsc70 was used as a loading control. Lanes 1–9 represent individual mice. *n*=3. (**b**) The protein amount of PHH3 from (**a**) was normalized to histone H3 levels (**a**) in order to quantify the transit amplifying cell marker of each genotype. Each column represents three genotypes presented as average fold change as related to K8^+/+^ control mice±SD, **P<0.01. (**c**) Frozen colonic sections were fixed with 4% PFA and immunostained with rabbit anti-PHH3 (green) and phalloidin recognizing F-Actin (red). Nuclei are presented in blue. The arrows point to examples of transit amplifying cells and representative images of *n*=3 mice are shown. L=lumen, M=muscle. Scale, 100 μm

The differentiation of colonic epithelial cells was clearly shifted towards secretory cell types, so the number of transit amplifying cells, from which the terminally differentiated cells originate, was investigated.^16^ Phosphohistone H3 (PHH3, marker of proliferative/ transit amplifying cells^34^) was significantly higher in both K8^−/−^ and K8^+/−^ mice colonic epithelium compared to K8^+/+^ when normalized to histone H3 (Figures 7a and b), indicating a dose-dependent increase in transit amplifying cells with reduced K8 levels (Figure 7a). Immunofluorescence staining confirmed the increased number of transit amplifying cells in the proliferative zone of the colonic crypts of K8^−/−^ and K8^+/−^ (Figure 7c). These observations indicate that the keratin dose-dependent colonic epithelial hyperproliferation^11^ is caused by an increased number of transit amplifying cells and a widened proliferative zone leading to longer crypts.^4^

## Discussion

**Figure 8:**
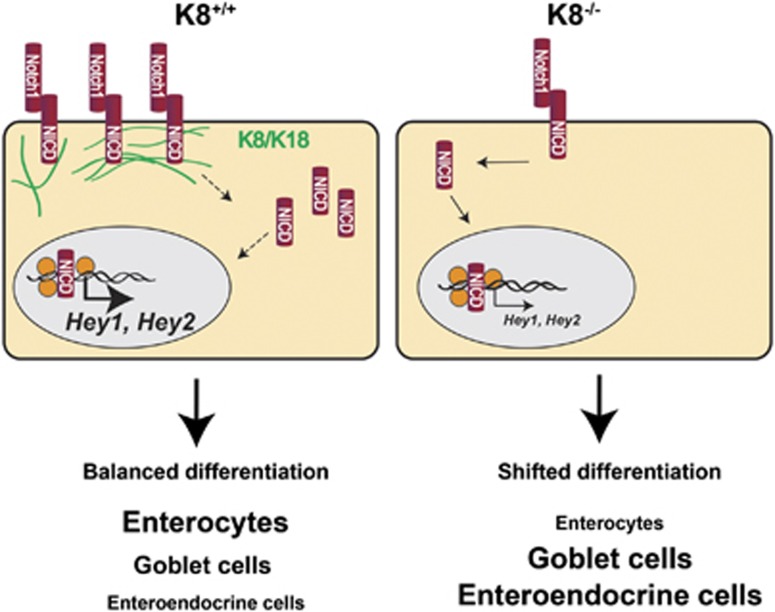
Summary of the impact of keratins on the Notch1 signalling pathway. K8/K18 bind Notch1, which increases NICD levels and enhances transcription of Notch1 target genes *Hey1* and *Hey2*. Therefore, the lack of K8 in the K8^−/−^ colon leads to decreased levels of FLN and NICD, and thus decreased target gene transcription. The lack of keratins leads to a Notch1-dependent shift in colonic epithelial cell differentiation with elevated number of goblet cells and EEC and a decreased number of enterocytes

We here describe a novel *in vivo* K8/K18–Notch1 interaction that has physiological importance for the differentiation of epithelial cells in the large intestine. We show with co-immunoprecipitation and PLA that K8 and K18 interact with Notch1 *in vivo* and *in vitro*. K8/K18 enhance Notch1 levels, promote downstream NICD activity and Notch1 target gene transcription. This association is supported by *in vitro* findings with CRISPR/Cas9 K8^−/−^ cells and MEFvim^−/−^, and *in vivo* findings where the loss of keratins in the colon leads to a dramatic decrease in Notch1 levels and signalling. In addition, the K8-dependent Notch1 phenotype can be rescued by re-expressing K8/K18 in K8^−/−^ cells. The K8 knockdown-induced reduction in Notch1 signalling causes a characteristic^19^ intestinal epithelial cell fate shift, with an increased number of colonic goblet cells, transit amplifying cells and EEC, and a decreased number of enterocytes (summarized in Figure 8). The K8^−/−^ phenotype is in line with the well-known effect of Notch inhibition, where blocking the cleavage of FLN to NICD by the gamma secretase inhibitor DAPT leads to goblet cell hyperplasia.^22, 35, 36^ The K8^+/−^ mouse colon has a partial intermediate proliferation and differentiation phenotype compared to K8^−/−^ and K8^+/+^ mice (Supplementary Table S1), in line with the milder effect on Notch1 levels seen after antibiotics treatment (Figures 3e and f). Although it is unclear whether the 50% decrease of K8 in K8^+/−^ mice is sufficient to significantly alter colonic Notch1 signalling, we here demonstrated in MEFvim^−/−^ cells a dose dependency of K8/K18 levels on the stability of NICD. To our knowledge, this is the first study showing that the structural and stress protector intermediate filament proteins participate in maintaining cell fate and differentiation through interaction with Notch1 affecting Notch1 activity and Notch1 target gene transcription.

The K8^−/−^ colon differentiation phenotype with more goblet cells is in accordance with the decreased Notch1 signalling activity and a shift in cell fate. However, the mucin mRNA levels appear to be compromised in the K8^−/−^, possibly indicative of problems in terminal goblet cell differentiation. This may be related to a previous description of abnormal containment of K8^−/−^ goblet mucus vacuoles.^4^ We cannot rule out that K8^−/−^ mice would not have a problem with terminal differentiation of colonic epithelial cell. As of note, deletion of K9 has been shown to lead to impaired terminal differentiation in epidermis,^37^ and K17 has been reported to have a role in the terminal differentiation in cervical epithelium.^38^ We further observe an elevated number of transit amplifying cells in colonic crypts after K8 deletion, which supports the previously described hyperproliferation phenotype,^4, 11, 12, 13^ and identifies the cell source of the increased proliferation in K8^−/−^ mice, in line with previous reports on hyperproliferation in the intestine.^39^ To that end, K19 was recently found to mark Lgr5-radioresistant cancer stem cells in the colonic epithelium,^40^ proposing a role for keratins in the crypt base compartment together with the Notch1 pathway as we here show that type I K19 is a similarly good partner to K8 as is K18 with respect to stabilizing NICD levels. Taken together, these data suggest that keratins have multifaceted roles in processes of tissue differentiation and turnover.

K8/K18 interact with Notch1, and the decreased NICD levels and signalling activity in the colon of the K8-knockout mice explain the observed goblet cell hyperplasia. Keratins are known to aid in protein localization, and intermediate filament proteins' mutations or deletion lead to mistargeting of membrane proteins.^4, 41, 42, 43^ As keratins do not affect Notch1 mRNA levels, FLN or NICD turnover, nor NICD degradation, it is conceivable that keratins could assist the transport of FLN to the plasma membrane. How keratins or other intermediate filament proteins do this is still elusive and needs further study. Post-translational modifications of keratins affect the binding to many keratin-associated proteins, including signalling intermediates, linker proteins and kinases.^44, 45^ Cytoplasmic keratins might, thus, prolong NICD activity by stabilizing and retaining a pool of NICD in the cytoplasm, facilitate Notch1 receptor cleavage, and/or affect FLN stability. Interestingly, keratins interact with 14-3-3, which was recently shown to affect nuclear translocation of Notch4.^46^ As the phosphodeficient mutant K8 S74A can bind and stabilize Notch1, but demonstrates reduced Notch1 target gene activation, the phosphorylation of K8 may have a role in some steps of this process. K8 S74 phosphorylation is a major dynamic K8 phosphorylation site in cell stress, cell division and apoptosis.^9, 47^ K8 S74 phosphorylation, which renders keratin filaments more soluble,^9^ could release NICD from keratin filaments and promote nuclear translocation and Notch1 target gene transcription.

A few other studies support a link between Notch1 and intermediate filament proteins. The expression of nestin, a marker for neural stem cells, is regulated by Notch1,^48^ and Notch1 activity as well as the expression of nestin decreases during differentiation.^49^ Astrocyte intermediate filament proteins regulate the Notch ligand Jagged to Notch1 signalling, and GFAP^−/−^Vim^−/−^ astrocytes show reduced endocytosis of Jagged1 with reduced Notch1 signalling in neuronal stem cells promoting neurogenesis.^50^ K14 siRNA knockdown in skin increase the levels of Notch1,^51^ and Notch1 downregulation decreases K13 and K15, but increases K17 levels.^52^ Further recent support for a keratin–Notch link is that Notch2 was decreased in K19-knockout mouse livers in the common bile duct ligation-injury model.^53^

In summary, the loss of keratins leads to a robust decrease in Notch1 which is linked to a shifted cellular differentiation in colonic epithelia. Keratins and Notch1 interact, leading to enhanced Notch1 stability and target gene transcription (Figure 8). These novel findings suggest that keratins are important regulators of Notch1 signalling activity and differentiation of colonic epithelial cells.

## Materials and Methods

### Experimental animals, sample collection and antibiotic treatment

K8^+/+^, K8^−/−^ and K8^+/−^ mice in the FVB/n background^12^ and of 4–6 months of age were used in this study. The mice were age- and sex-matched and genotyped as previously described.^12, 54^ After being killed by CO_2_ inhalation, colonic epithelial cells were isolated by scraping the lumen side of the colon with an ice-cold glass slide,^55^ and total colon samples were collected from the 1-cm central part of the colon. Samples were used for western blot, immunoprecipitation, RT-PCR, and high salt extraction and immunostainings. For depleting the colonic microflora, K8^+/+^, K8^−/−^ and K8^+/−^ mice were treated with the broad-spectrum antibiotics vancomycin and imipenem (Hospira, IL, USA) administered in drinking water at 68 mg/kg body weight/day for 8 weeks starting at 18–19 days postnatally.^13^ The drinking water containing antibiotics was changed three times a week. Control mice received normal drinking water. Animal experiments were approved by and carried out in accordance with the National Animal Experiment Board and conformed to the regulations set by The Finnish Act on Animal Experimentation.

### Cell culture, transfections, cycloheximide and MG132 assays

Colorectal cancer Caco-2 cells, and vimentin knockout MEFvim^−/−^ cells (mouse embryonic fibroblasts that lack vimentin and any other cytoplasmic intermediate filament proteins; a gift from Professor John Eriksson, Åbo Akademi University^56^) were grown in DMEM supplemented with 10% fetal calf serum, 2 mM L-glutamine and 100 U/ml penicillin/streptomycin. Both MCF7 and HEK 293-expressing FLN (HEK 293 FLN) cells were cultured in DMEM (Sigma-Aldrich, MO, USA) supplemented with 10% fetal calf serum, 2 mM L-glutamine, 100 U/ml penicillin and 100 μg/ml streptomycin. For HEK 293 FLN cells, 10 μg/ml puromycin was added to DMEM. All cells were cultured at 37 °C in a 5% CO_2_ atmosphere. K8-knockout Caco-2 cells and corresponding control Caco-2 cells with normal K8 levels were generated by CRISPR/Cas9 technology as previously described.^26^ K8^−/−^ and K8^+/+^ CRISPR/Cas9 cells were rescued by transfecting 2 μg human K8 and human K18 DNA, or with NICD-GFP-Flag with Lipofectamine 2000 (Invitrogen, CA, USA) for 24 h following the manufacturer's protocol. For transfection of MEFvim^−/−^ cells, cells were washed and re-suspended in 400 μl Opti-MEM (Invitrogen) and placed in electroporation cuvettes (BTX, CA, USA) with 10 μg DNA (human K8, K8 S74A, K18, K19, PCDNA3.1, NICD-GFP-Flag, FLN and ΔE Notch1). The cells were electroporated with Gene Pulser (Bio-Rad, CA, USA) at 975 μF, 260 V and incubated at 37 °C for 24–48 h, and transfection efficiency was 50–60%. For Notch1 stability assays, MEFvim^−/−^ cells, or K8^−/−^ CRISPR/Cas9 and K8^+/+^ CRISPR/Cas9 Caco-2 cells were treated with 10 μg/ml cycloheximide (Sigma-Aldrich, MO, USA) for 0–6 h, and the proteasome was inhibited with 20 μM MG132 (Santa Cruz, TX, USA) for 12 h.

### Immunoprecipitation

Mouse distal (DC) and proximal (PC) epithelium was isolated for immunoprecipitation by scraping the lumen-side layer of colon with a cold glass slide and lysed in 400 μl lysis buffer (25 mM Hepes (pH 8.0), 100 mM NaCl, 5 mM EDTA, 0.5% Triton X-100, 20 mM *β*-glycerophosphate, 20 mM para-nitro-phenyl phosphate, 100 μM ortovandante, 0.5 mM phnylmethanesulfonylfluoride, 1 mM or 200 mM dithiothreitol, complete mini protease cocktail (Roche, Switzerland)). MEFvim^−/−^ and HEK FLN 293 cells were transfected and washed in PBS for immunoprecipitation. The colon epithelium, HEK 293 FLN and MEFvim^−/−^ cells were lysed in 400 μl lysis buffer. The precleared lysate was incubated with rabbit anti-K8 and rabbit anti-K18 (Professor John Eriksson, Åbo Akademi University^57^), or mouse anti-K18 L2A1 (Professor Bishr Omary, University of Michigan), or goat anti-full length Notch c-20 (Santa Cruz) antibodies on shaker in RT for 30 min after which 40 μl 50% diluted protein-G/Sepharose (GE Healthcare, UK) was added to each sample for 16 or 4 h at 4 °C. The samples were centrifuged, washed four times in TEG buffer (20 mM Tris-HCl (pH 7.5), 1 mM EDTA, 10% Glycerol), dissolved in Laemmli sample buffer and analysed by SDS-PAGE and western blot. The *in vivo* and *in vitro* immunoprecipitation samples are not normalized to each other. The *in vitro* immunoprecipitation in Figure 1b was quantified by subtracting the non-antibody signal from the immunoprecipitation samples and dividing the result with the input signal.

### Proximity ligation assay

Caco-2 cells were cultured on coverslips and fixed at –20 °C for 10 min with methanol and 1 min with acetone for PLA analysis. Primary antibodies used were rabbit anti-full-length Notch c-20 (Santa Cruz), mouse anti-K8 (Progene, Germany). Mouse anti-Cox1 (Santa Cruz) was used as a negative control known not to bind to Notch1.^58^ A PLA kit (mouse and rabbit, Duolink, Sigma-Aldrich, MO, USA) was used where plus and minus probes were mixed into antibody dilution buffer and incubated for 1 h at 37 °C. After the samples were washed, samples were incubated with ligation buffer together with ligase for 30 min at 37 °C. Samples were thereafter amplified with amplification buffer, washed and mounted with mounting media containing DAPI (included in the PLA kit). The stained cells were analysed with Zeiss LSM780 confocal microscope and quantification was performed using particle analysis in Fiji software (NIH, MD, USA).

### SDS-PAGE and western blot

Protein samples were homogenized on ice in homogenization buffer (0.187 M Tris-HCl, 3% SDS, 5 mM EDTA, pH 6.8).^59^ Protein concentration was measured with BSA protein assay reagent kit (Thermo-Scientific, IL, USA), and samples were normalized and diluted in Laemmli sample buffer, heated at 95 °C for 3–5 min and 10 μg protein/sample was loaded in each well on 7–12% SDS-polyacrylamide gels. Prestained molecular weight markers (Bio-Rad) were loaded on each gel, and the molecular weights of the analysed bands are indicated on the right side on every blot/panel in the figures where kD refers to kilodalton. Rabbit anti-*β*-actin (Cell Signaling, MA, USA), rabbit anti-phosphohistone H3 (Cell Signaling), rabbit anti-histone H3 (Abcam, UK), rat anti-Hsc70 (Stressgen Bioreagents, MI, USA), rabbit anti-synaptophysin (Abcam), mouse anti-villin (Abcam), rabbit anti-full length Notch c-20 (Santa Cruz), rat anti-Troma I (Developmental Studies Hybridoma Bank, IA, USA), rabbit anti-K8 (273) and rabbit anti-K18 (275; John Eriksson, Finland) and mouse anti-cytokeratin 4.62 (K19, Sigma-Aldrich, CA, USA) were used as primary antibodies. Anti-rabbit IgG (Promega, WI, USA), anti-rat IgG (GE Healthcare and Cell Signaling) and anti-mouse IgG (GE Healthcare) were used as secondary antibodies. ECL (GE Healthcare) and ECL plus (Perkin Elmer, MA, USA) were used for detection of blots on X-ray films (Super RX, Fuji Corporation, Japan). Individual bands were analysed and normalized to the loading controls Hsc70 or actin using the ImageJ Software (NIH, MD, USA).

### RNA isolation and RT-PCR

Total colon RNA was isolated (from colon scrapings and total colon samples) using an RNA kit (Macherey Nagel, Germany), and the RNA quality was analysed in 1% agarose gel. A total of 1 μg of each RNA sample was synthesized by reverse transcription to cDNA using a transcription kit (Promega). Target genes were amplified using specific primers (Supplementary Table S2) and KAPA probe Fast ABI Prism qPCR mix (Kapa Biosystems, MA, USA). qPCR was performed with StepOnePlus Real-Time PCR system (Applied Biosystems, CA, USA). Gene expression levels were normalized to *β-Actin*. Each cDNA was tested in triplicates, and the amplification was analysed using 1% agarose gel. For CRISPR/Cas9 K8^+/+^ and K8^−/−^ Caco-2 cell mRNA analysis, total RNA was isolated, reverse transcribed to cDNA and qPCR performed as above, but gene expression levels were normalized to *GAPDH*.

### Alcian blue and periodic acid Schiff staining

Formalin-fixed paraffin-embedded colon was sectioned, deparaffinized and incubated in 1% Alcian blue or periodic acid (Sigma-Aldrich, CA, USA) for 30 min highlighting the mucus in goblet cells. Schiff reagent (Merck, NJ, USA) was used to stain the background after periodic acid staining (PAS) after which the sections were washed, incubated in Myers hemalum (Merck) and dehydrated. For Alcian blue, after washing, the sections were counterstained in nuclear fast red solution for 5 min, washed and dehydrated with 95% ethanol. Sections were cleared in xylene and mounted with mounting medium. Alcian blue or PAS-positive cells were quantified by dividing the number of positive goblet cells in a colonic crypt with the total amount of cells in the colonic crypts. Sections from three separate mice were stained for Alcian blue, and the cells from 10 crypts per mouse were quantified so that 30 crypts per genotype were quantified.

### Immunofluorescence and immunohistochemical staining

Colonic 7-μm sections of PC and DC were fixed in 1–4% paraformaldehyde (Sigma-Aldrich. CA, USA). Human Caco-2 cells were cultured and grown as described above and fixed at −20 °C for 10 min with methanol and for 1 min with acetone. Cells of tissues were stained as previously described.^59^ Primary antibodies used were rat anti-Troma I (Developmental Hybridoma Bank, NIH, MD, USA), rabbit anti-K8 (273) and rabbit anti-K18 (275), rabbit anti-full length Notch c-20 (Santa Cruz), mouse-anti Notch1 (A6; Thermo Scientific), rabbit anti-Hrt1 (Hey1, Santa Cruz), rabbit anti-phosphohistone H3 (Cell Signaling) and rabbit anti-synaptophysin (Abcam). Secondary antibodies used were donkey anti-rabbit conjugated with Alexa Fluor 488 (Invitrogen) and goat anti-rat conjugated with Alexa Fluor 546 (Invitrogen). Draq5 (Cell Signaling) or DAPI (Invitrogen) were used to stain DNA, and Phalloidin (Invitrogen) to stain F-Actin. ProLong gold antifade reagent (Invitrogen) was used for mounting coverslips. Leica TCS SP5 confocal microscope with dry objective HC Plan Apo × 20/0.70 NA (Figure 6h and 7c) and a photomultiplier tube was used for image detection at room temperature using the Leica LAS AF acquisition software. Zeiss LSM780 confocal microscope with × 63/1.2 water objective was used for image detection (Figures 1d, 4e, 5, Supplementary Figures S2). Zeiss Zen image software was used for image analysis. Heatmap images (Figure 6h) from confocal microscopy data were created with the BioImageXD software.^60^ All images in the individual panels were acquired with the same settings and adjusted for brightness and contrast identically using Photoshop CS5 (Adobe, CA, USA).

Immunohistochemistry staining on paraffin-embedded colon sections was performed at the Department of Pathology, University of Turku Hospital. Sections were fixed in 4% PFA, incubated with rabbit anti-cleaved Notch1 (Abcam) primary antibody and secondary antibody conjugated to HRP. Sections were analysed with the Panoramic 250 Slide Scanner and Pannoramic Viewer was used for image acquisition using × 20 (Supplementary Figure S3) and × 40 (Figure 3d) settings.

### Statistical analyses

Cell culture and animal experiments were repeated three or more times. One-way Anova with Bonferroni's *post hoc* test or two-tailed Student's *t*-test (GraphPad Prism Software, CA, USA) were used to determine the statistical difference. *P* values of *P<0.05, **P<0.01, ***P<0.001 were considered as significant with 95% confidence interval.
