# Supplementary figures and images for: Keratins regulate colonic epithelial cell differentiation through the Notch1 signalling pathway
Source: Cell Death Differ. 2017 May 5;24(6):984–96. doi: 10.1038/cdd.2017.28 (PMC5442467; doi:10.1038/cdd.2017.28)

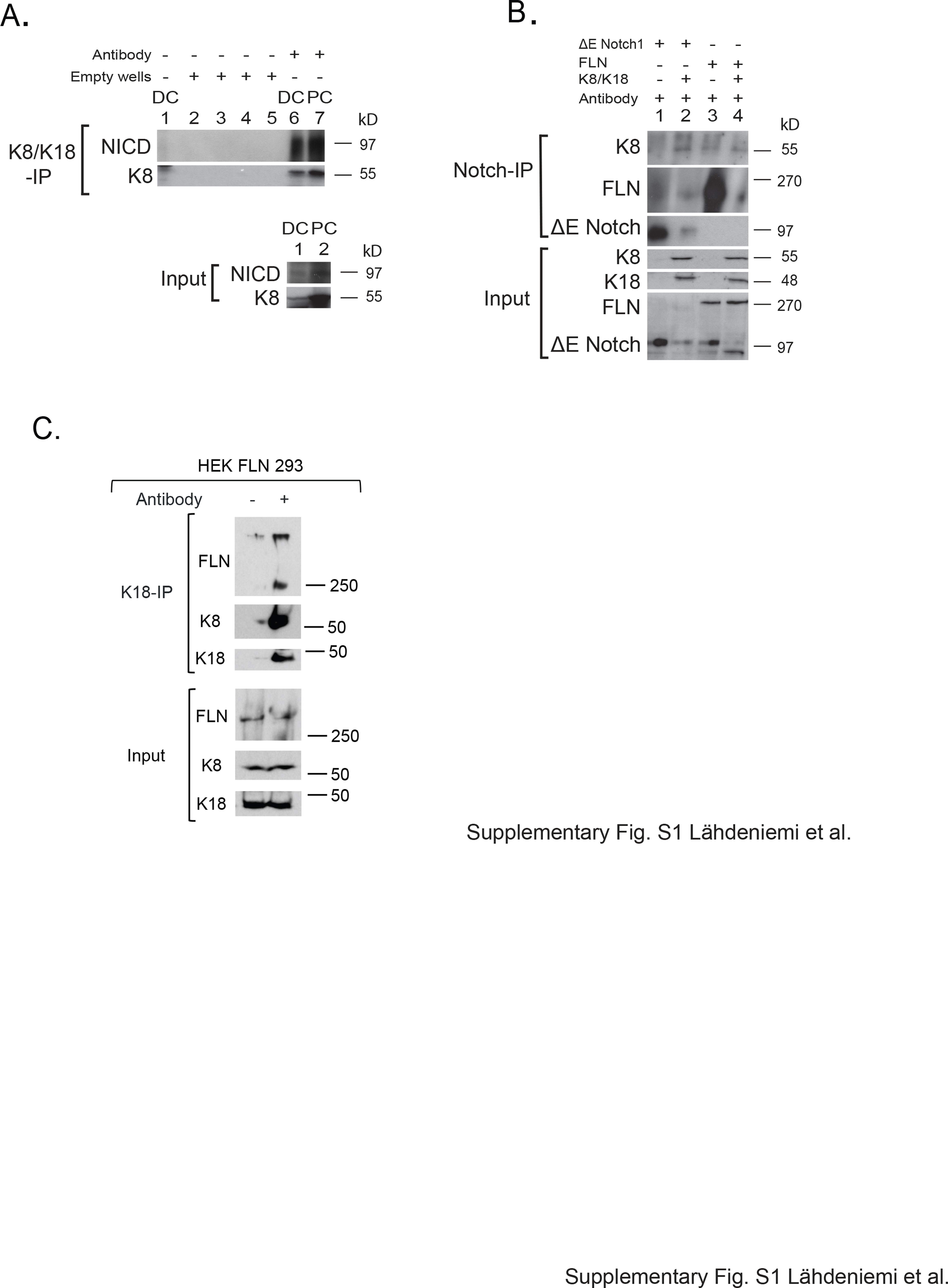

Supplement: Supplementary Figure 1 [file cdd201728x1.tif]

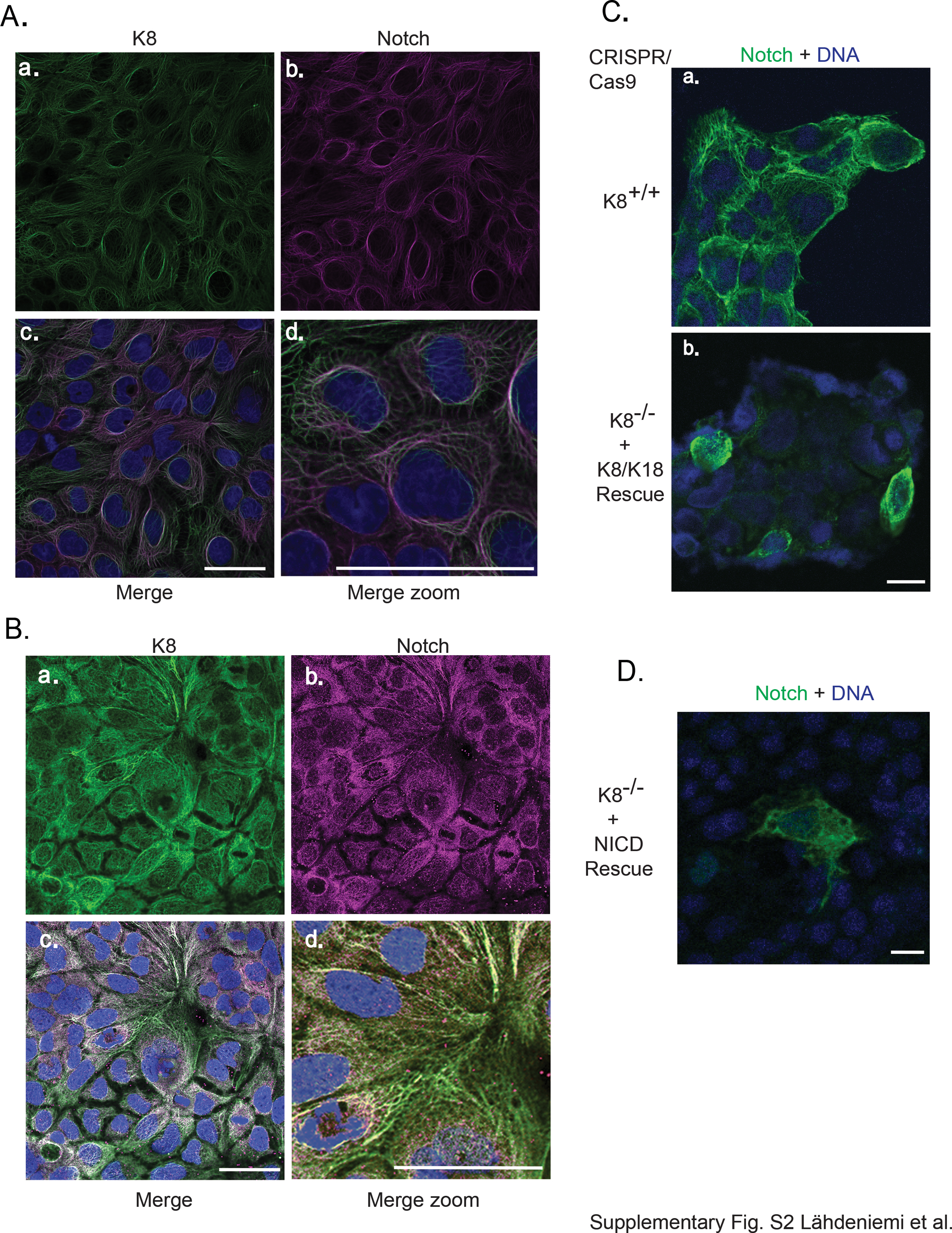

Supplement: Supplementary Figure 2 [file cdd201728x2.tif]

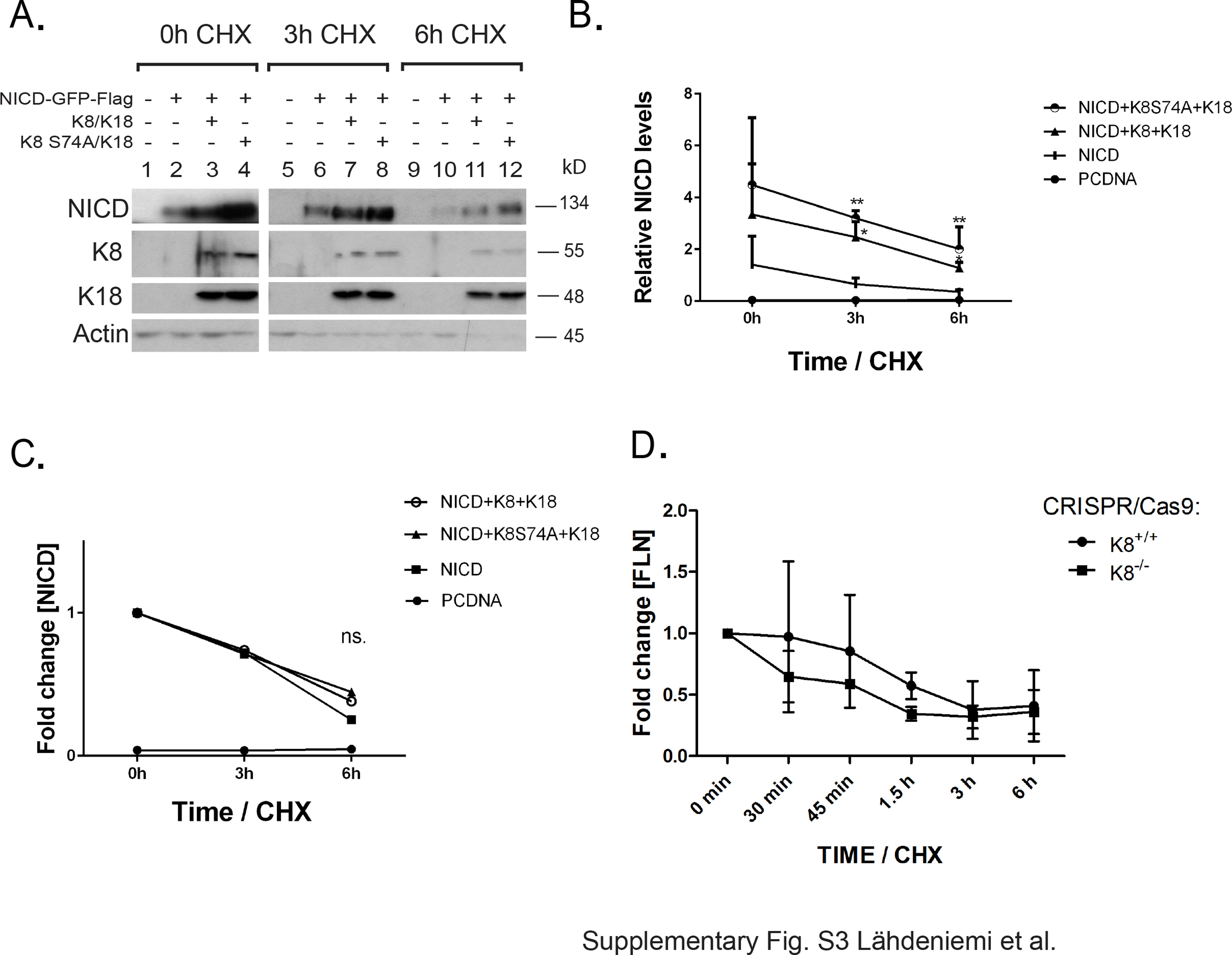

Supplement: Supplementary Figure 3 [file cdd201728x3.tif]

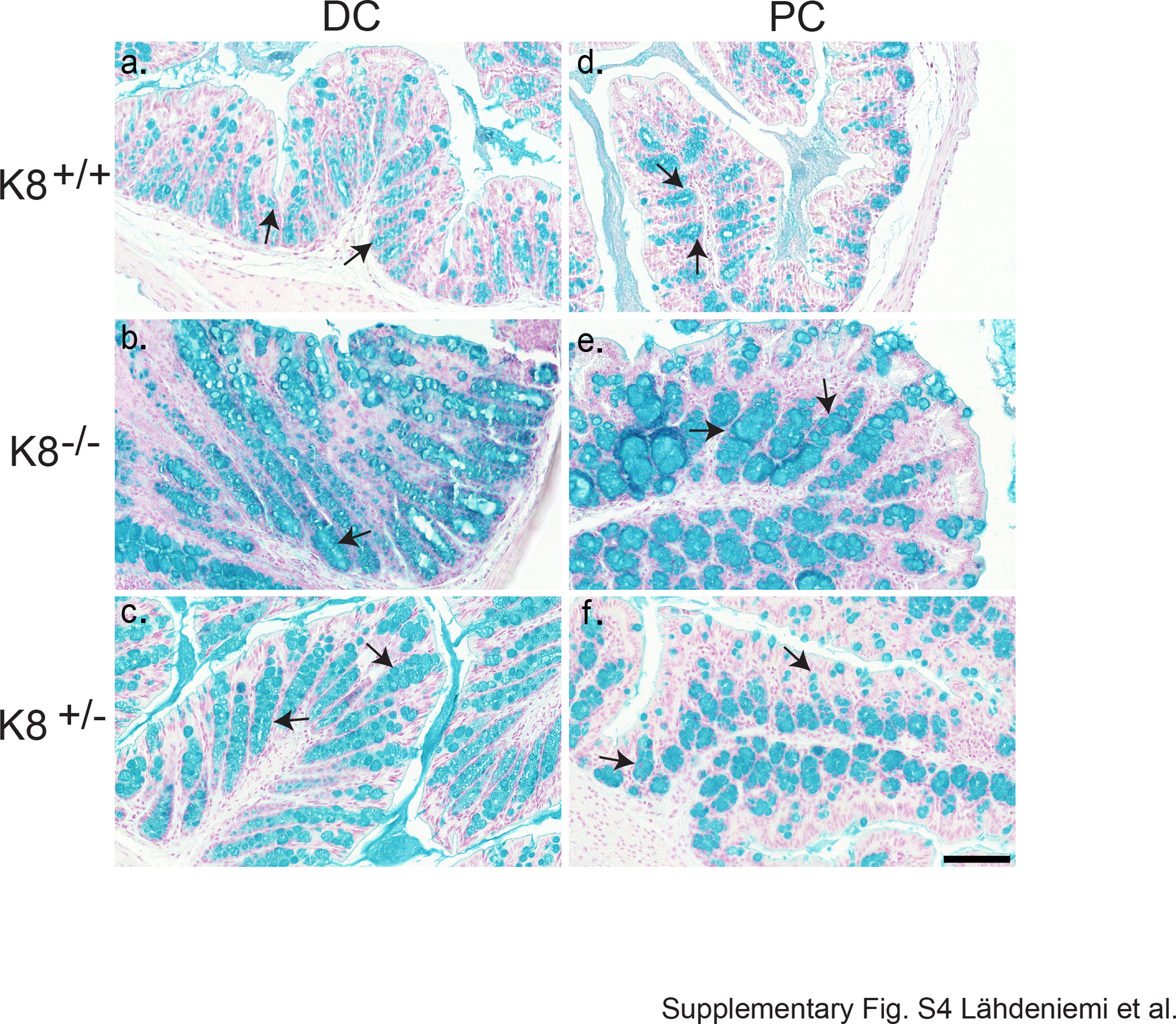

Supplement: Supplementary Figure 4 [file cdd201728x4.tif]
