# Supplementary material for: Keratins regulate colonic epithelial cell differentiation through the Notch1 signalling pathway
Source: Cell Death Differ. 2017 May 5;24(6):984–96. doi: 10.1038/cdd.2017.28 (PMC5442467; doi:10.1038/cdd.2017.28)
Supplement: Supplementary Tables 1 and 2 [file cdd201728x6.pdf]

Lähdeniemi et al. Supplementary Figures and Tables

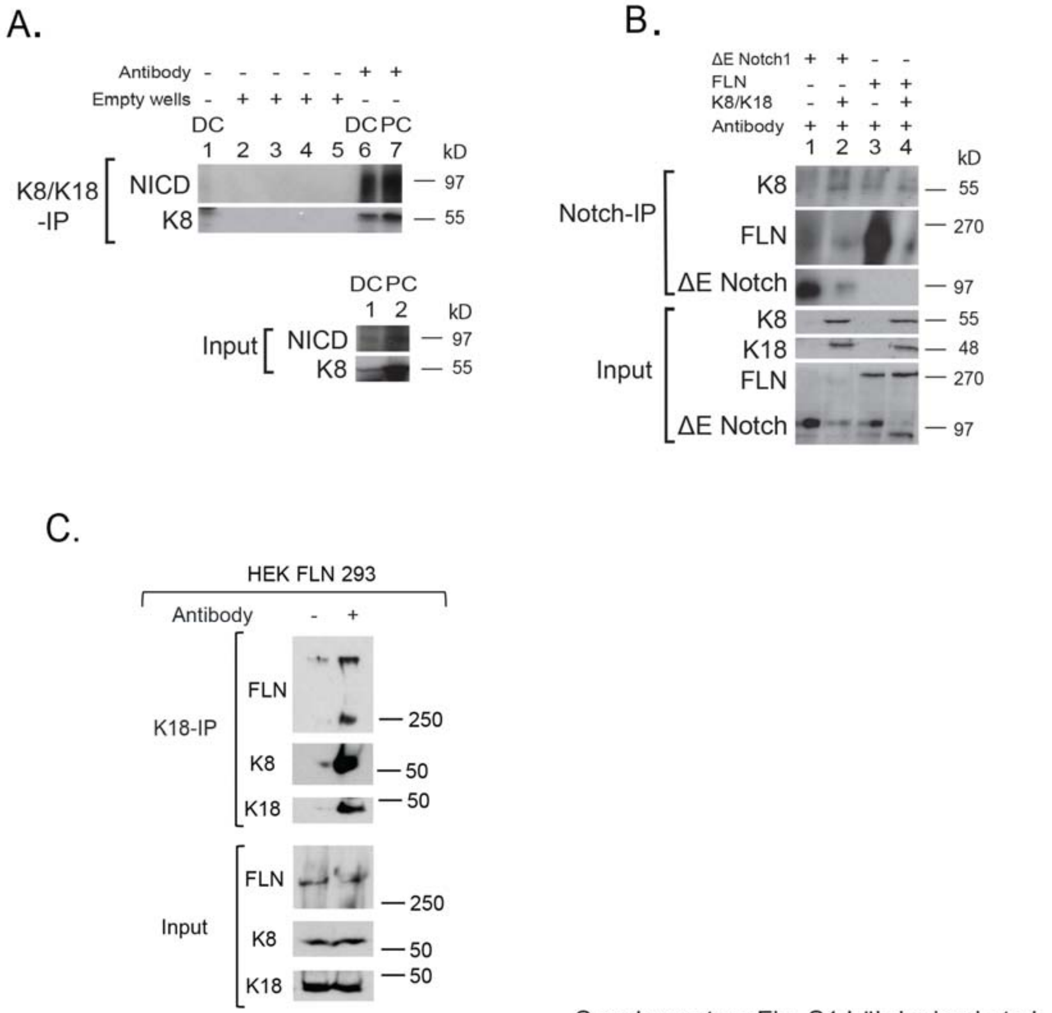

Supplementary Fig. S1 Lähdeniemi et al.

Supplementary Figure S1. **K8, K18 and Notch1 co-immunoprecipitate.** (**A)** Proximal (PC) and distal (DC) parts of the colon epithelium were isolated by scraping and homogenized with immunoprecipitation lysis buffer. For K8/K18-immunoprecipitation (IP), the lysates were precleared with protein-G/Sepharose beads and incubated over night with beads and K8/K18 antibodies. The immunoprecipitates were analyzed with SDS-PAGE and immunoblotting with the indicated antibodies. Input samples were collected before the immunoprecipitation. Separate sample (-antibody) was prepared from the DC sample which was treated the same way as the other samples exept that no antibody was added. n=4. (**B**) MEFvim$^{-/-}$ cells were transfected with the indicated plasmids and the samples were collected 24 hours after transfection and lysed for Notch immunoprecipitation (IP) as described in Fig. 1B. The precipitates were precleared and incubated 16 hours with beads and goat anti-full length Notch c-20 antibody recognizing all Notch forms. The immunoprecipitates were analyzed with SDS-PAGE and immunoblotting with the indicated antibodies. For control samples see Fig. 1B. **(C)** HEK FLN 293 cells were lysed and the precleared samples were immunoprecipitated (K18-IP) against K18 with G-sepharose beads and the L2A1 anti-human K18 antibody for 4 hours at 4°C. Input samples (Input) were collected before the immunoprecipitation. Samples lacking antibody during immunoprecipitation (Antibody -) were used as negative controls. The samples were separated by SDS-PAGE and immunoblotted with indicated anti-Notch1 c-20, K8 and K18 antibodies.

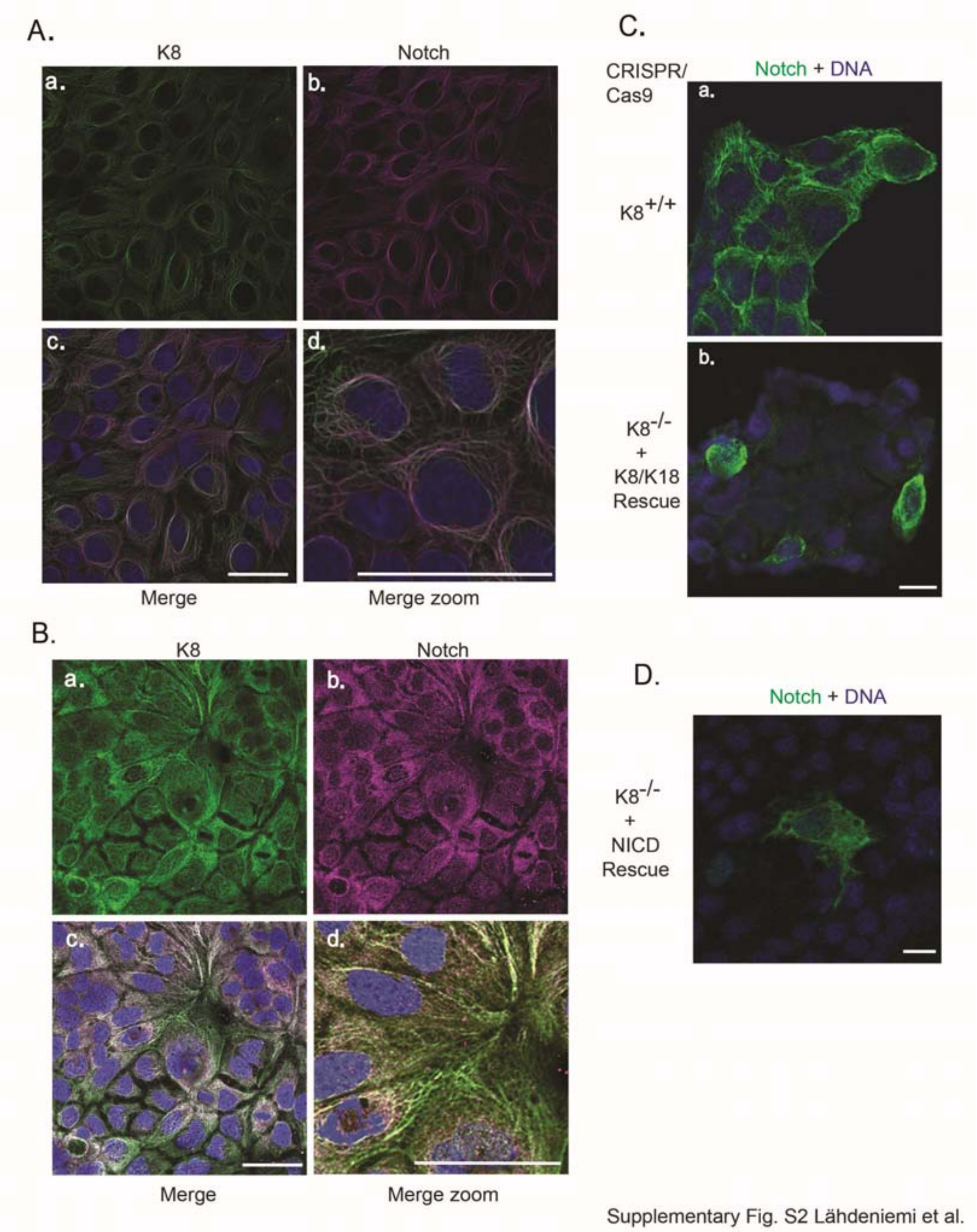

Supplementary Figure S2. **K8 co-localizes partly with Notch1 in cultured epithelial cells, and K8/K18 or NICD rescue of K8-/- cells rescues Notch1 levels.** (**A**)-(**B**) Caco-2 (A) cells and MCF7 cells (B) were fixed with methanol and acetone at -20°C. (A) Caco2 cells were co-immunostained for K8 (a, c, d, green; rat anti-K8) and for Notch1 (b, c, d; magenta; rabbit anti-full length Notch1 c-20). In (B) MEF7 cells were co-immunostained for K8 (a, c, d, green; rabbit anti-K8) and for Notch1 (b, c, d; magenta; mouse anti-xx Notch1 A6). Bars in A and B = 50 µm. In **(C)** Notch1 (green) was stained alone in CRISPR/Cas9 $K8^{+/+}$ Caco-2 cells (a), and in CRISPR/Cas9 $K8^{-/-}$ Caco-2 cells where K8/K18 had been re-expressed (b, Rescue), n=3. **(D)** CRISPR/Cas9 $K8^{-/-}$ Caco-2 cells overexpressing NICD-Flag-GFP were fixed with methanol and acetone at -20° and stained for Notch1 (green) and nuclei (blue) alone Bar= 20 µm. n=2. Nuclei are presented in blue.

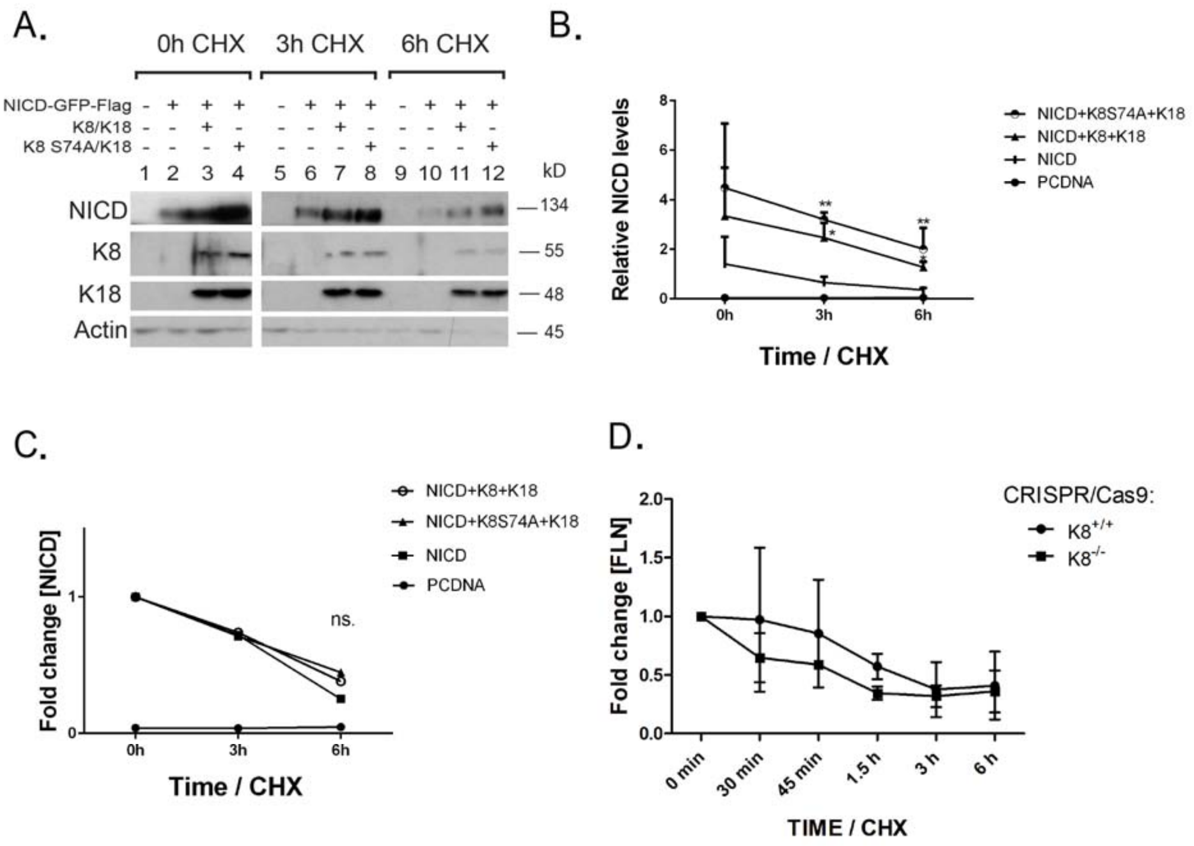

Supplementary Fig. S3 Lähdeniemi et al.

Supplementary Figure S3. **Similar degradation of NICD and FLN after translation blockage with and without keratins.** (**A**) MEFvim$^{-/-}$ cells were transfected by electroporation with the indicated plasmids. 12 hours after transfection cells were treated for 0, 3 and 6 hours with 10 µg/ml cycloheximide (CHX). Cells were analyzed by immunoblotting. Actin was used as a loading control. Lanes 1, 5 and 9 represents transfection with an empty plasmid (PCDNA3.1). The white space indicates adjacent gels/blots that were processed in parallel with samples derived from the same experiment. n=3. (**B**) The protein amount of NICD from 0, 3 and 6 hours CHX treated MEFvim$^{-/-}$ cells (A) was normalized to the loading control actin and the relative NICD amounts are presented as average ± SD. n=3. (**C**) The protein levels of NICD in (D) at 3 and 6 hours of CHX treatment was normalized to the basal levels of NICD at 0 hours to determine the degradation speed of NICD. n=3. (**D**) CRISPR/Cas9 Caco-2 K8$^{+/+}$ and K8$^{-/-}$ cells were treated for 0-6 hours with 10 µg/ml CHX. Cells were analyzed by immunoblotting, normalized to actin and the FLN protein levels were quantified and presented as average ± SD, n=3.

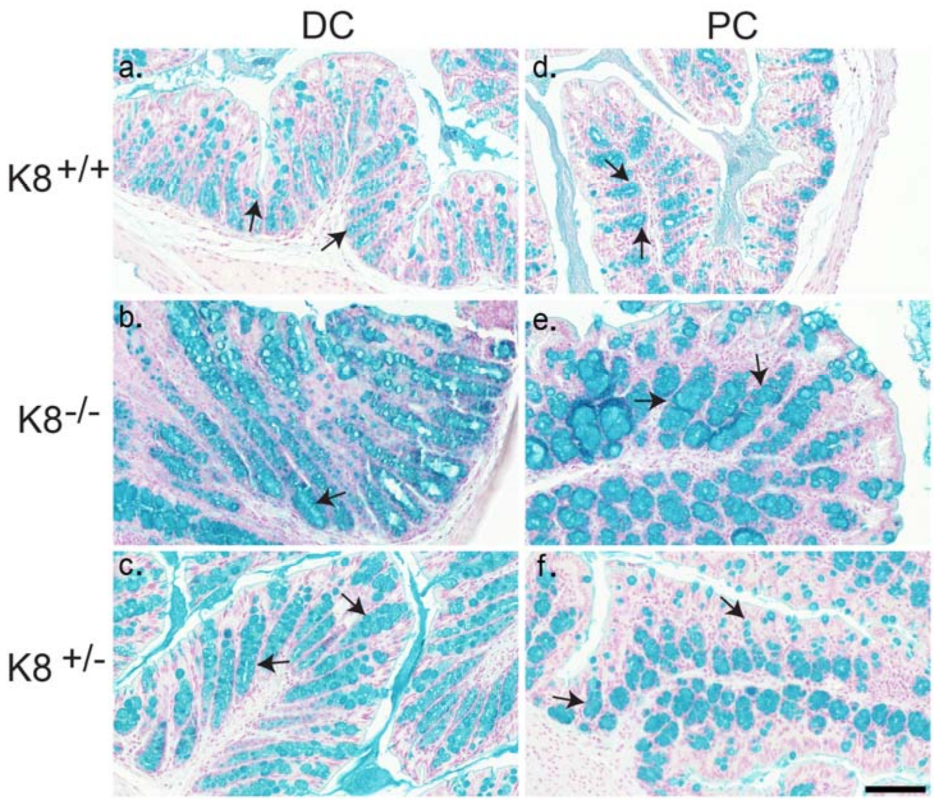

Supplementary Fig. S4 Lähdeniemi et al.

Supplementary Figure S4. **The number of goblet cells is increased in K8$^{-/-}$ and K8$^{+/-}$ DC and K8$^{-/-}$ PC.** Formalin-fixed and paraffin embedded DC and PC from K8$^{+/+}$ (a, d), K8$^{-/-}$ (b, e) and K8$^{+/-}$ (c, f) were incubated in 1% Alcian blue highlighting the mucus in goblet cells. The Alcian blue staining was quantified (in Fig. 6D) by dividing the number of alcian blue positive goblet cells to the total amount of cells in the colonic crypts. The arrows point to examples of mucus positive goblet cells. n=3. Scale, 100 µm.

Lähdeniemi et al, Supplementary Tables

Suppl. Table S1. Summary of the mouse colonic phenotypes caused by full ($K8^{-/-}$) or partial ($K8^{+/-}$) K8-deletion on epithelial cell differentiation and Notch signaling in vivo compared to wildtype mice ($K8^{+/+}$). + - ++++ indicate relative amounts of cells, proteins or mRNA ranging from none (-) and few (+) to much (++++). NS= not significant. TA, transit amplifying cells; EEC, enteroendocrine cells; FLN, full length Notch1; NICD, notch intracellular domain; Abx, antibiotics treatment. *) Note $K8^{+/-}$ colon FLN was not statistically different from $K8^{+/+}$ colon after antibiotics treatemnt by One-way Anova, but gave a p-value of p=0.05 when compared with student's t-test.

Geno-type	K8 levels	TA-cells	Goblet cells	EEC	Entero-cytes	FLN, NICD	FLN (Abx)	Hey1, Hey2
$K8^{+/+}$	++++	+	+	+	+++	+++	+++	+++
$K8^{-/-}$	+/-	+++	+++	++++	+	+	+	+
$K8^{+/-}$	++	++	++	++	++NS	+++	++*)	++NS

Suppl. Table S2. **Primers used for RT-PCR.**

Primers for mouse genes:

Gene	Forward primer	Reverse primer
β-Actin	5'-TGG CTC CTA GCA CCA TGA AGA-3'	5'-GTG GAC AGT GAG GCC AGG AT- 3'
CA2	5'-GGG GAT ACA GCA AGC ACA AC-3'	5'-GAC TGC CGG TCT CCA TTG- 3'
Mucin1	5'-CTG TTC ACC ACC ACC ATG AC-3	5'-TTG GAA GGG CAA GAA AAC C-3'
Mucin2	5'-CTT CTG TGC CAC CCT CGT -3'	5'-TTC GGG ATC TGG CTT CTT T-3'
Notch1	5'-CTG GAC CCC ATG GAC ATC-3	5'-AGG ATG ACT GCA CAC ATT GC-3'
Hey1	5'-ACC ATC GAG GTG GAA AAG G-3'	5'-CTT CTC GAT GAT GCC TCT CC-3'
Hey2	5'-GTG GGG AGC GAG AAC AAT TA-3'	5'-GTT GTC GGT GAA TTG GAC CT-3'

Primers for human genes:

Gene	Forward primer	Reverse primer
**Hey1**	5'-CGAGCTGGACGAGACCAT-3'	5'-GAGCCGAACTCAAGTTTCCA-3'
**GAPDH**	5'-GTTCGACAGTCAGCCGCATC-3'	5'-GGAATTTGCCATGGGTGGA-3'
